# The Pig: A Relevant Model for Evaluating the Neutrophil Serine Protease Activities during Acute *Pseudomonas aeruginosa* Lung Infection

**DOI:** 10.1371/journal.pone.0168577

**Published:** 2016-12-16

**Authors:** Claire Chevaleyre, Mickaël Riou, Déborah Bréa, Clarisse Vandebrouck, Céline Barc, Jérémy Pezant, Sandrine Melo, Michel Olivier, Rémy Delaunay, Olivier Boulesteix, Patricia Berthon, Christelle Rossignol, Julien Burlaud Gaillard, Frédéric Becq, Francis Gauthier, Mustapha Si-Tahar, François Meurens, Mustapha Berri, Ignacio Caballero-Posadas, Sylvie Attucci

**Affiliations:** 1 Infectiologie et Santé Publique (UMR 1282 ISP), INRA, Université Tours, Nouzilly, France; 2 Plateforme d'Infectiologie expérimentale (UE-1277 PFIE), INRA, Nouzilly, France; 3 INSERM, Centre d'Etude des Pathologies Respiratoires, UMR 1100, Tours cedex, France; 4 Laboratoire Signalisation et Transports Ioniques Membranaires, Université de Poitiers, Centre National de la Recherche Scientifique, Poitiers cedex, France; 5 Département des Microscopies (Plateau technologique Analyse des systèmes Biologiques), Université François-Rabelais, Tours cedex, France; 6 BioEpAR, Oniris, Nantes Atlantic National College of Veterinary Medicine, Food Science and Engineering La Chantrerie, Nantes Cedex 3, France; Hospital for Sick Children, CANADA

## Abstract

The main features of lung infection and inflammation are a massive recruitment of neutrophils and the subsequent release of neutrophil serine proteases (NSPs). Anti-infectious and/or anti-inflammatory treatments must be tested on a suitable animal model. Mice models do not replicate several aspects of human lung disease. This is particularly true for cystic fibrosis (CF), which has led the scientific community to a search for new animal models. We have shown that mice are not appropriate for characterizing drugs targeting neutrophil-dependent inflammation and that pig neutrophils and their NSPs are similar to their human homologues. We induced acute neutrophilic inflammatory responses in pig lungs using *Pseudomonas aeruginosa*, an opportunistic respiratory pathogen. Blood samples, nasal swabs and bronchoalveolar lavage fluids (BALFs) were collected at 0, 3, 6 and 24 h *post-*insfection (p.i.) and biochemical parameters, serum and BAL cytokines, bacterial cultures and neutrophil activity were evaluated. The release of proinflammatory mediators, biochemical and hematological blood parameters, cell recruitment and bronchial reactivity, peaked at 6h p.i.. We also used synthetic substrates specific for human neutrophil proteases to show that the activity of pig NSPs in BALFs increased. These proteases were also detected at the surface of lung neutrophils using anti-human NSP antibodies. *Pseudomonas aeruginosa-*induced lung infection in pigs results in a neutrophilic response similar to that described for cystic fibrosis and ventilator-associated pneumonia in humans. Altogether, this indicates that the pig is an appropriate model for testing anti-infectious and/or anti-inflammatory drugs to combat adverse proteolytic effects of neutrophil in human lung diseases.

## Introduction

Inflammatory lung diseases like ventilator-associated pneumonia (VAP), chronic obstructive lung disease (COPD) and cystic fibrosis (CF) all involve a massive recruitment of neutrophils to the lungs [1–3]. These immune cells clear pathogenic agents using several oxidative and proteolytic pathways [4, 5]. During this process neutrophils secrete large amounts of neutrophil serine proteases (NSPs: neutrophil elastase (NE), protease 3 (Pr3) and cathepsin G (cat G)) that overwhelm the capacity of endogenous antiproteases to control their activity, ultimately leading to the destruction of lung tissue [6–8].

*Pseudomonas aeruginosa (P*. *aeruginosa)*, a bacterium that rarely infects human lungs unless the host immune system has been impaired [9], is one of the main pathogens found in CF, COPD and VAP [10–12]. A wide array of transgenic mice models has been developed to study chronic lung inflammatory diseases, such as CF [13]. Mice models are also used to evaluate the role of neutrophils in the progress of acute and chronic neutrophil-associated lung diseases that frequently involve *Pseudomonas aeruginosa (P*. *aeruginosa)* infection [14–18]. Despite the important advances made with these models, several research groups have acknowledged that they have inherent limitations [18–21]. There are important differences in the lifespans of mice and humans as well as in their airway architecture that make it impossible to study the chronicity of lung diseases. Thus rodent models cannot reproduce the complex features of human diseases like CF [19–21]. Differences in the substrate specificities of mouse and human NSPs [22] also seriously complicate testing and validating anti-inflammatory therapies that target NSPs.

Porcine lungs share many anatomical, histological, biochemical, and physiological features with those of humans [23]. We have previously shown that porcine and human neutrophils behave similarly *in vitro*, both releasing NSPs and neutrophil extracellular traps (NETs) in response to *P*. *aeruginosa* infection [24]. Pig NSPs also have the same substrate specificities and similar immunochemical properties as their human homologues. Thus, they can be inhibited by the human natural inhibitors α1-proteinase inhibitor (α1-Pi) and α1-antichymotrypsin (ACT), which makes the pig a relevant model for developing drugs that target human NSPs.

We have developed an experimental model of *P*. *aeruginosa* lung infection and acute inflammation in normal pigs in which to evaluate the neutrophilic response in terms of neutrophil recruitment to the lungs, and the production of proinflammatory mediators, biochemical and hematological parameters, the proteolytic activities of secreted NSPs, NETs formation and bronchial reactivity.

## Materials and Methods

### Animal preparation

All experiments were conducted in accordance with the guidelines of the European Council Directive (86/609). The experimental procedures were approved by the Loire Valley ethical review board (CEEA VdL, committee number n°19, number 2011-11-03). Pigs were kept in Biosafety Level 2 confined housing (9 m^2^/pig) that was cleaned daily throughout the experimental procedure. They had access to a standard grain-based diet (Sanders) and water *ad libitum*. Social and material (balls) enrichment was provided to maintain pig welfare.

The physical condition of all animals was monitored twice per day. Animal welfare was determined by assessing the following parameters: general condition, feeding, body temperature, heart rate, respiratory rate, mucous colour, faeces, nasal discharge, coughing and weight. A protocol was set up for using humane endpoints. Pigs were sacrificed when they showed at least 3 of the following major clinical signs of pain and distress: hyperthermia (>41°C), prostration, anorexia, diarrhea and/or vomiting, significant weight loss, tissue necrosis, biting. No animal became unexpectedly ill or died during the procedures.

A total of twelve healthy 10-week-old large white WT pigs weighing 30 ± 5 kg were obtained from the (ANSES) Ploufragan-Plouzané (Ploufragan, France). They were obtained from a herd that is seronegative for all common diseases (S1 Table). Pigs were infected with Pseudomonas aeruginosa under anesthesia to minimize pain and distress. They were sedated with intramuscular Ketamine (20 mg/kg; Imalgene®, Mérial, France) and Xylazine (2 mg/kg; Rompun®, Bayer, Germany) and then anesthetized with isoflurane (Vetflurane®, Virbac, France). The trachea of each pig was intubated and the animal ventilated mechanically with a Fabius® Tiro® Ventilator (Dräger, Telford, USA). The ventilator settings were: volume controlled mode, tidal volume = 8–10 mL.kg^-1^, positive end-expiratory pressure = 5 cm H_2_O, respiratory rate = 15 breaths.min^-1^, and inspiratory/expiratory ratio = 0.5, 50% oxygen in air. For sacrifice, pigs were anesthetized as above, given i.v. heparin (Choay®, 25 000 UI/5 mL; Sanofi-Adventis, France), and exsanguinated before collection of post-mortem samples.

### Bronchial inoculation with *Pseudomonas aeruginosa*

A total of 6 pigs were each inoculated with 70 mL of a suspension of *P*. *aeruginosa* strain PAK [25] (8 x 10^6^ cfu/mL) in sterile PBS 1X without calcium or magnesium via the bifurcation of the right and left main bronchus using an esophageal probe. Control pigs (n = 6) were inoculated with 70 mL of sterile PBS 1X without calcium or magnesium. The pigs were ventilated mechanically until they recovered from the anesthesia. The whole procedure lasted less than 20 minutes.

### Follow-up and samples collection

**Fig 1:**
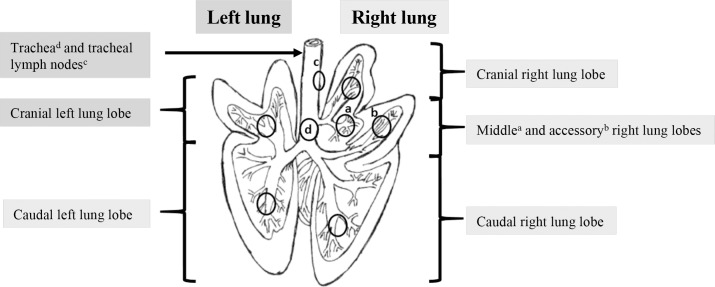
Anatomy of the pig lung. Diagram from C.L. Pavaux [26]. The black circles correspond to areas where samples were taken on each pig (cranial left lung lobe, caudal left lung lobe, cranial right lung lobe, middle right lung lobe, accessory right lung lobe and caudal right lung lobe), trachea (d: bronchial crossroads) and tracheal lymph nodes.

Body temperature, respiratory and heart rates were monitored at 0, 6 and 24 h. Blood samples were collected for blood cell counts, biochemical parameters and cytokine assays at 0, 3, 6 and 24 h. Nasal swabs and bronchoalveolar lavage (BAL) fluid were collected at 0, 6 and 24 h p.i.. BAL was collected by instilling and re-aspirating two 50-mL aliquots of sterile PBS without calcium or magnesium with an esophageal probe inserted through the tracheal intubation. The lungs, tracheal lymph nodes (Fig 1) and spleen were collected at 6 h and 24 h p.i. for bacteriological and histological analysis.

### Biochemical parameters

The serum biochemical parameters (alanine aminotransferase (ALAT), aspartate aminotransferase (ASAT), bilirubin, glucose, creatinine, and urea) were measured using Select-6V rings with the M-ScanII Biochemical analyzer (Melet Schloesing Laboratories, France). C-reactive protein (CRP) was assayed with CRP Pig ELISA kits Abnova® (Walnut, CA, USA): normal reference CRP value at least 6 ng/mL.

### Evaluation of cytokines and chemokine in serum and BAL

Tumor necrosis factor (TNF)-α, interleukin (IL)-6 and IL-8 were measured in serum and BAL supernatant using Abcam® Pig ELISA kits (Paris, France) according to the manufacturer’s recommendations.

### Blood cell count

Blood cells were counted with a MS9-5 Hematology Counter® (digital automatic hematology analyzer, Melet Schloesing Laboratories, France).

### Bronchial reactivity

Strips of pig bronchi (4 mm width and 15 mm length) were placed in Krebs solution as previously described for rat, pig and human tissues [27–29]. Cumulative concentration-response relationships for the relaxant effect of salbutamol (Sigma-Aldrich, St Quentin Fallavier, France) were determined in bronchial rings following stable contraction. The relaxant effect was expressed as percentage contraction of the carbachol-constricted bronchial rings. IC50 was calculated as the drug concentration inducing a half-maximal dilatation (or inhibition of contraction).

### Histopathology findings

Sections (5 μm) of 4% formalin-fixed and paraffin-embedded samples were stained with haematoxylin and eosin (H&E). The epithelial goblet cells were stained with periodic acid-Schiff (PAS) reagent. All samples were examined with an Eclipse 80i microscope equipped with a DXM 1200C digital camera (Nikon Instruments, Europe, Amsterdam, Netherlands) and NIS-Elements D Microscope Imaging Software. The severity of pneumonia was graded as follows: Level 1: mild response with healthy tissue in most sections. Level 2: inflammatory response involving nearly half of the area of tissue sections, level 3: strong, diffuse inflammatory response involving most of the section areas, with only a small area of healthy tissue. Level 4: intense response with inflammatory foci leading to a partial loss of alveolar spaces (lung hepatization). Level 5: acute inflammatory response with loss of most alveolar spaces [30, 31].

### Bacteriological analysis

Nasal swabs (qualitative cultures) and BAL fluid, lung lobes, trachea, thoracic lymph nodes and spleen (quantitative cultures) were cultured on Cetrimide selective medium (Oxoid, Dardilly, France) using standard laboratory methods [32].

### Flow cytometry analysis of cell populations in BAL

The cell populations in BAL were characterized by their specific surface markers [33] as described in [24]. Briefly, BAL fluid was centrifuged at 1,000 g for 10 min. The resulting cells were suspended in PBS (10^5^ in 100 μL PBS), transferred to low-binding polypropylene 96-well plates (Corning; Avon, France) and analysed with a MACSQuant^®^ Analyser flow cytometer (Miltenyl Biotec); data for at least 10,000 events were recorded.

### Confocal microscopy

Neutrophil elastase was detected using anti-human NE antisera raised in rabbits [24]. Approximately 3 x 10^5^ cells were seeded onto Superfrost slides and treated as described previously [24]. Briefly, primary rabbit anti-peptide antibodies specific for each human protease were diluted 1/200 (anti-NE) and bound antibodies were detected with an anti-IgG coupled to FluoProbes^®^-488 (Interchim, Montluçon, France). DRAQ5™ (Interchim, Montluçon, France) (10 μM) was used to detect dsDNA. Samples were examined with an Olympus FV 500 confocal microscope (Olympus, Rungis, France). Purified peripheral blood neutrophils were activated by incubation with *P*. *aeruginosa* at a MOI (multiplicity of infection) of 1:20 for 1 h at room temperature before evaluation as described in Brea *et al* [24].

### Immunoblotting

Neutrophil elastase was detected by immunoblotting using monoclonal anti-human NE antibodies (Abcam®, Paris, France). The proteins in aliquots (6 μL) of BAL fluid supernatants corresponding to 1.2 x 10^5^ BAL cells taken 6h p.i. and 1.4 x 10^4^ cells from control animals were separated by SDS-PAGE (12%) and transferred to nitrocellulose membranes. These membranes were incubated with the primary antibody diluted 1:1000. Bound antibodies were detected with a goat anti-mouse antibody coupled to horseradish peroxidase (Sigma-Aldrich, St Quentin Fallavier, France) diluted 1:5000. Immunoreactivity was visualized by enhanced chemiluminescence with an ECL detection kit (GE Healthcare Europe) using a MF-ChemiBIS 3.2 reader (DNR Bio Imaging System).

### Measurement of serine protease activities in BAL

The protease activities in 150 μL of BAL supernatant were measured [24] using the FRET substrates (Genecust, Dudelonge, Luxemburg) optimized for human NSPs: Abz-TPFSGQ-EDDnp for cat G (EC 3.4.21.20) [34], Abz-VADCADYQ-EDDnp for Pr3 (EC 3.4.21.76) [35] and that designed for mouse NE (EC 3.4.21.37), Abz-QPMAVVQSVPQ-EDDnp [22].

### Statistical analysis

Results are expressed as means ± SEM of at least 3 replicates. Statistical analyses were performed using GraphPad Instat and GraphPad Prism versions 5.0 (GraphPad Software, La Jolla, CA). Biochemical parameters, bronchial reactivity, total and differential WBC counts and neutrophil serine protease activity were analyzed using non-parametric Mann-Whitney U tests. A two-way analysis of variance (ANOVA) model was used to differentiate between the effects of time and infection on the cytokine and bacteriological measurements. Post-hoc mean separation was performed using Bonferroni-adjusted pairwise comparisons. Differences in the severity of the histological lesions of control and infected groups were determined using a contingency chi-squared test. Differences between groups were considered significant when p<0.05.

## Results

### Clinical follow-up and biochemical parameters

Body temperature was significantly elevated 6 h p.i. in the *P*. *aeruginosa* inoculated group but had returned to normal by 24 h (Table 1). ASAT was significantly increased in the control group 6 h post-infection, and returned to normal after 24 h (Table 1). There were no significant differences in the other clinical and biological parameters evaluated in *P*. *aeruginosa-*inoculated and control pigs (Table 1).

**Table 1:** Clinical follow-up, blood biochemistry and C-reactive protein in pigs after *P*. *aeruginosa* infection.

Biological Parameter	Before infection		6 h post-infection		24 h post-infection	
	Control	Infected	Control	Infected	Control	Infected
**Temperature (°C)**	39.0±0.1	38.9±0.1	39.2±0.2	40.1±0.2^*^	39.0±0.1	39.2±0.03
**Heart rate (bpm)**	115. 3±5.8	127.0±4.9	132.3±7.3	129.0±5	116.0±9.2	123.3±4.6
**Respiratory rate**	53.33±3.3	54.66±4.9	54±4.3	64.33±10.1	53.33±11.5	73.33±18.6
**Haematocrit (%)**	32.0±5.0	32.9±1.7	37.5±3.0	35.1±2.9	36.1±1.2	29.1±1.7
**Bilirubin (mg/L)**	2.9±0.3	3.3±0.9	2.5±0.1	2.5±0.2	1.6±0.2	2.8±0.4
**ASAT (U/L)**	41.0±7.4	38.5±3.0	74.6±10.3^*^	43.3±6.9	34.3±13.4	23.7±10.7
**ALAT (U/L)**	40.1±3.0	45.2±4.0	53.7±3.1	48.3±4.4	48.0±2.1	41.7±4.2
**Glucose (g/L)**	0.96±0.03	1.07±0.07	1.31±0.07	1.25±0.05	1.18±0.16	1.09±0.16
**Urea (g/L)**	0.12±0.02	0.23±0.03	0.42±0.03	0.39±0.03	0.18±0.04	0.25±0.05
**Creatinine (mg/L)**	12.0±0.7	11.7±0.7	15.2±1.4	12.6±0.6	13.8±0.6	10.6±0.5
**CRP (μg/mL)**	87.2±14.5	71.9±11.1	105.4±18.0	92.0±8.0	146.0±45.3	231.0±41.5

Control and infected groups were compared using the Mann-Whitney U test at each time point.

* indicates p<0.05 between control and infected group.

**Fig 2:**
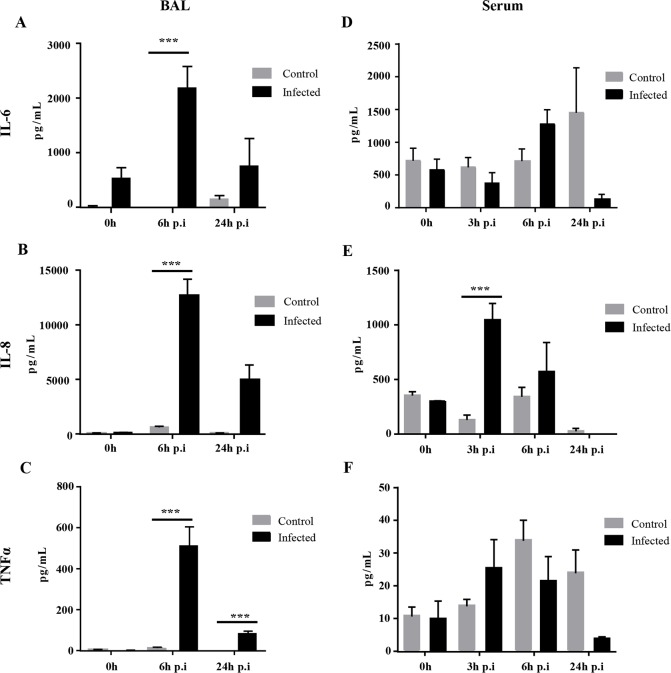
Cytokine concentrations in the BAL and serum of pigs after *P*. *aeruginosa* infection. A. IL-6, IL-8 and TNF-α in BAL fluid. B. IL-6, IL-8 and TNF-α in serum. Data were analysed by two-way ANOVA followed by Bonferroni's *post hoc* test. Data are means ± S.E.M. * indicates p<0.05. *** indicates p<0.001.

The concentrations of TNF-α, IL-8 and IL-6 were increased in the BALs of infected pigs 6 h p.i.. IL-8 and IL-6 had returned to normal at 24 h (Fig 2A–2C). Serum IL-6 did not change significantly in either control or infected pigs (Fig 2D). The IL-8 concentration in the serum of infected pigs was significantly elevated 3 h p.i., remained high at 6 h p.i. and decreased at 24 h p.i. (Fig 2E). However, the serum TNF-α concentration did not vary significantly (Fig 2F). The cytokine concentrations in the control pigs remained unchanged.

### Bronchial reactivity

**Fig 3:**
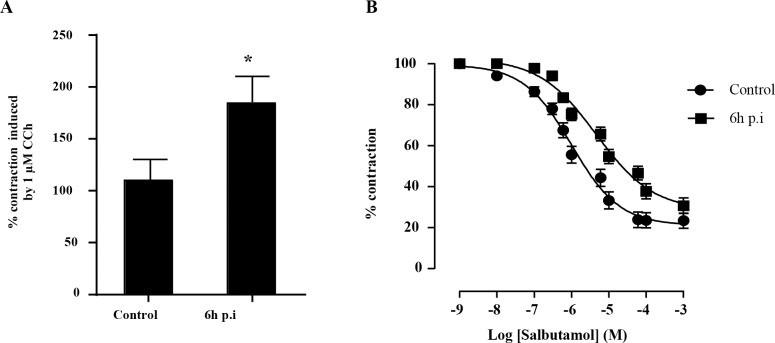
Effect of *P*. *aeruginosa* infection on the reactivity of porcine bronchial smooth muscle cells. A. Influence of *P*. *aeruginosa* infection on the constriction of porcine bronchial rings induced by 1μM carbachol 6 h after infection. B. Concentration dependence of the dilatation of carbachol-contracted bronchial rings induced by salbutamol in control and 6 h after *P*. *aeruginosa* infection. Control and infected groups were compared using the Mann-Whitney U test. Data are means ± S.E.M. * indicates p<0.05. A sigmoidal dose-response curve was used to generate the IC50.

Carbachol (1μM) induced the infected bronchi removed 6 h p.i. to contract nearly twice as much as control bronchial segments (Fig 3A). Adding salbutamol to the organ bath caused carbachol-constricted porcine bronchial rings to relax in a concentration-dependent manner. The salbutamol concentration producing half-maximal relaxation (IC50) in 16 control bronchial rings was 2.24 ± 1.01 μM. (Fig 3B) while the IC50 for infected bronchial rings was greater (23.98 ± 1.10 μM) (Fig 3B). Carbachol caused bronchial samples taken 24 h p.i. to contract nearly twice as much as control bronchial segments (data not shown). Cumulative addition of salbutamol to the organ bath also induced a concentration-dependent relaxation of porcine bronchial rings (data not shown).

### Histological findings

**Fig 4:**
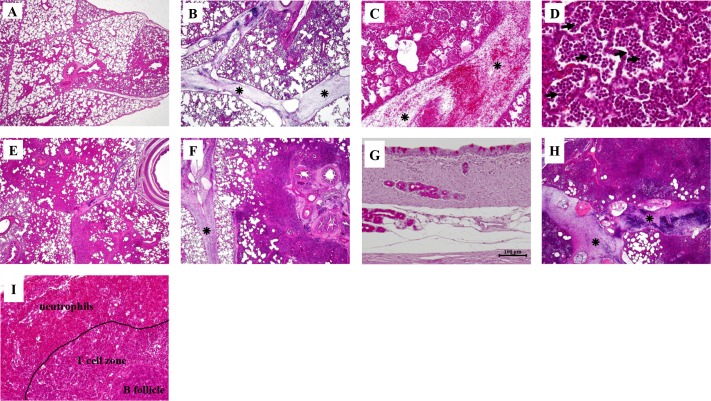
Histology of pig lung tissue sections and trachea sections taken 6 h and 24 h after *P*. *aeruginosa* infection. Sections of pig lung tissue were stained with haematoxylin & eosin. Degrees of inflammation are given in Table 2. **A**. Section of lung (right middle lobe; x 20) from a control PBS-instilled pig. **B**. *P*. *aeruginosa*-induced level 1 inflammatory response 6 h after infection showing blood cells in the dilated interlobular septa (*) and in lung tissue (left cranial lobe; x 20). **C**. Blood cells in the dilated interlobular septa (left cranial lobe) (x 100). **D**. Leukocytes, mainly neutrophils, in the alveolar spaces (left cranial lobe) (x 400). **E**. Inflammatory response (level 3) appearing as early as 6 h after infection with small areas of healthy tissue and occlusion of alveolar spaces by neutrophils and red blood cells (right cranial lobe; x 20). **F**. Foci of inflamed areas around a bronchus and blood vessels 24 h p.i., indicating a level 4 inflammatory response (right middle lobe; x 20). **G**. Section of trachea sampled 6 h p.i. (x 200). **H**. Inflammatory response 24 h p.i., with a great increase of leukocytes and red blood cells leading to intense dilation of the interlobular septa, occlusion of most alveolar spaces, and hepatisation of the lung tissue (left cranial lobe; x 20). **I**. Neutrophils in the tracheal lymph nodes 24 h p.i. (x 200).

Blood cells were observed in the lungs, from cranial to caudal lobes, at 6 h p.i. (Fig 4A–4E). The inflammatory response was characterized by dilated interlobular septa (Fig 4B and 4C) and infiltration of cells, mainly neutrophils and red blood cells, into the alveolar spaces (Fig 4D and 4E). Samples taken 24 h p.i. showed large inflammatory foci in the trachea and around the bronchi and blood vessels (Fig 4F and 4G) and occlusion of alveolar spaces with neutrophils, leading to hepatization of the lung tissue (Fig 4H). Neutrophils had also infiltrated the draining tracheal lymph nodes by 6 h and 24 h p.i. (Fig 4I). The pneumonia of infected pigs was more severe than that of the controls (Table 2). We also observed more mucous goblet cells in the tracheae of *P*. *aeruginosa*-infected pigs than in the PBS controls (S1 Fig).

**Table 2:** Histological evaluation of the *P*. *aeruginosa-*induced inflammatory response in the lungs of control and infected pigs.

	6 h				24 h			
	None or mild (%)	Medium (%)	Severe (%)	*p* value	None or Mild (%)	Medium (%)	Severe (%)	*p* value
**Control**	83.33	16.66	0	<0.0001	61.11	38.89	0	0.0034
**Infected**	5.55	61.11	33.34		22.22	33.33	44.45	

This table shows the percentages of animals classified according to lesion severity. None or mild: histological score lower than 2; Medium: histological scores 2 and 3; Severe: histological score higher than 3. *p* values were calculated using the contingency chi-squared test.

### Bacteriological analyses

**Fig 5:**
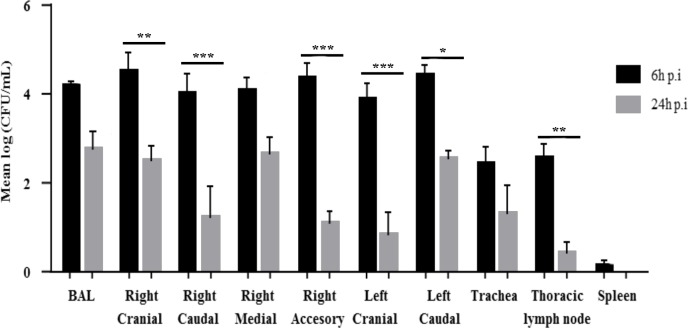
Bacterial loads in the BAL fluid, lung lobes, trachea, thoracic lymph nodes and spleen of pigs infected with P. aeruginosa. Pigs were inoculated with 70 mL of an 8 x 10^6^ cfu/mL suspension of P. aeruginosa PAK strain. Controls were obtained by inoculating 70 mL of 1X sterile PBS. Data were analysed by two-way ANOVA followed by Bonferroni’s *post hoc* test. Data are means ± S.E.M. * indicates p<0.05. ** indicates p<0.01. *** indicates p<0.001.

Analysis of nasal swabs and BAL fluid showed that all pigs were free of *Pseudomonas spp*. before the experimental infection. The concentrations of *P*. *aeruginosa* in the BAL and all the lung samples were elevated (≥ 10^4^ cfu/mL) 6 h p.i. (Fig 5). *P*. *aeruginosa* was also found in the trachea and tracheal lymph nodes (≤ 10^3^ cfu/mL) and to a lesser extent in the spleen (Fig 5). The concentrations of *P*. *aeruginosa* had significantly decreased at 24 h p.i. in the right cranial, right caudal, right accessory, left cranial and left caudal lung lobes and in the thoracic lymph nodes (Fig 5). We found no *P*. *aeruginosa* in the control pigs at any time during the experiment.

### Total and differential white blood cell counts in the blood and BAL

**Fig 6:**
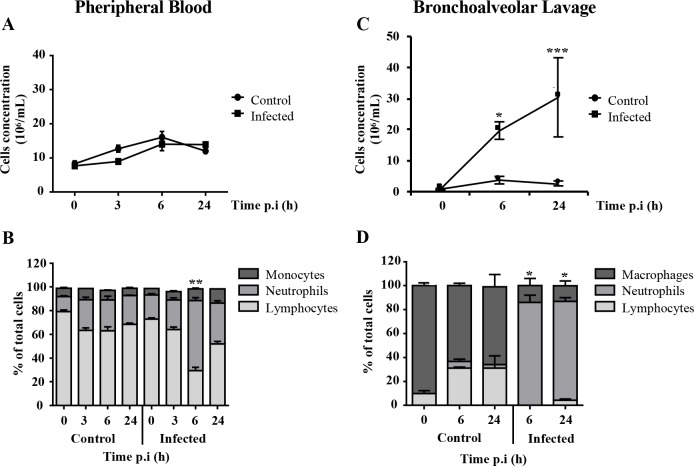
Total white blood cell counts and cell profiles in the peripheral blood and BAL fluid of pigs at different times after *P*. *aeruginosa* infection. A. Total white blood cells (WBC) in peripheral blood. B. Profile of the WBC populations in peripheral blood. C. Total WBC in BAL fluid. D. Profile of the WBC populations in BAL fluid. Total WBC and cell profiles of the control and infected groups were compared using the Mann-Whitney U test for each time point. Data are means ± S.E.M. * indicates p<0.05. ** indicates p<0.01. *** indicates p<0.001.

The leukocyte populations in the blood did not show significant differences between infected and control groups (Fig 6A). Differential cell counts showed that lymphocytes were the predominant leukocytes in the blood of the control pigs throughout the experiment (Fig 6B). Although results for the infected pigs were similar, the lymphocyte population in the blood of infected pigs dropped significantly and the neutrophil numbers peaked at 6 h p.i. (Fig 6B). BAL analysis showed that the total number of leukocytes was higher in infected pigs than in the controls (Fig 6C). The neutrophil concentration in the BAL fluid taken from infected pigs was significantly increased at 6 h and remained high at 24 h (Fig 6D).

### Neutrophil serine proteases in BAL fluid

**Fig 7:**
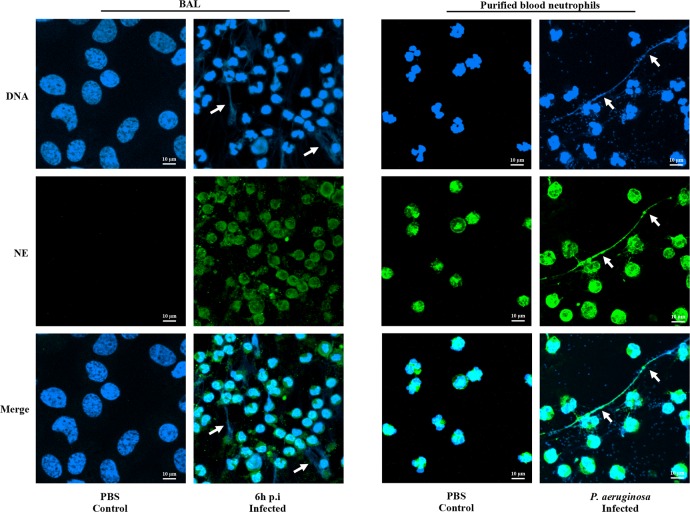
Neutrophil serine proteases in BAL fluids of pigs infected with *P*. *aeruginosa* and purified blood neutrophils. Confocal microscopy showing DNA (blue) and elastase (NE; green). Arrows show NET filaments.

Neutrophil elastase was visualized by confocal microscopy at the surface of BAL neutrophils collected from *P*. *aeruginosa* infected pigs at 6 h p.i. (Fig 7) while no labeling was observed in control BAL fluid samples (Fig 7). NETs were also present in BAL fluids 6 h p.i. (Fig 7). Similar NET-like structures and neutrophil elastase were observed in the extracellular milieu of *P*. *aeruginosa-*activated purified blood neutrophils (Fig 7).

**Fig 8:**
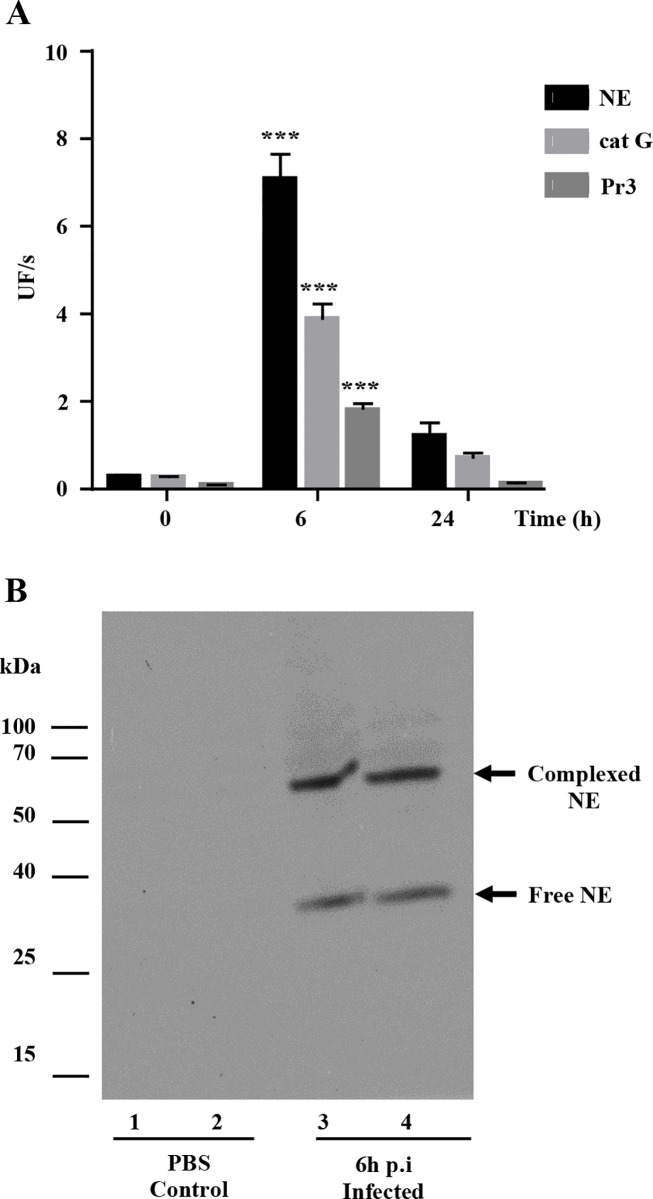
Peptidase activity of neutrophil serine proteases from the BAL fluid of pigs infected with *P*. *aeruginosa*. A. Peptidase activities of NE, Pr3 and cat G (means ± SEM; n = 6). Increased protease activity were analysed using the non-parametric Mann-Whitney U test. *** indicates a significant increase in enzyme activity (p<0.001). B. Western blotting of neutrophil elastase showing free protease (25 kDa) and an irreversible complex with serpins (63 kDa).

The activities of NE, cat G and Pr3 in the soluble fraction of BAL from infected pigs taken 6 h p.i. were much higher than in the PBS-treated controls, but decreased rapidly 24 h p.i. (Fig 8A). We ensured that no other class of proteases cleaved the NSP substrates using inhibitors of metalloproteases (10 mM EDTA), cysteine proteases (100 μM E64) and aminopeptidases (100 μM Bestatin). Western blotting analysis of BAL fluid supernatants showed the presence of free NE and irreversible complexes (Fig 8B).

## Discussion

Although murine models are some of the main tools of biomedical research, recent data has raised questions about the relevance of these animals for studies of inflammatory diseases and their implications for humans [36, 37]. This has prompted a search for new pre-clinical models [23]. Pigs have been shown to be good experimental models of *Pseudomonas aeruginosa* infections in VAP [38, 39]. Inoculating the lungs of pigs with *P*. *aeruginosa* caused increases in pro-inflammatory cytokines and triggered pneumonia in ventilated pigs. However, the functions of neutrophils and NSPs, the major players in the inflammatory response to *P*. *aeruginosa*, have received little attention. We have previously shown that *P*. *aeruginosa* stimulates purified pig peripheral blood PMNs to secrete NSPs and NETs *in vitro*, as observed in humans [24]. These data all indicated that the pig would be a suitable model for evaluating the neutrophil response to *P*. *aeruginosa* infection. The findings of this *in vivo* study using a pig model of *P*. *aeruginosa* lung infection confirm this suitability.

Inoculating the lungs of WT pigs with *P*. *aeruginosa* induces an acute predominantly local inflammatory response. This response is characterised by bronchial contraction, a transient increase in pro-inflammatory cytokines (IL-8, IL-6 and TNF-α), intense neutrophilia, NETosis and the secretion of massive amounts of NSPs that results in lung damage. In contrast, most of the biochemical parameters analysed as well as the inflammatory cytokines and bacteraemia remained low. These findings indicate the importance of a local immune response in the lungs rather than a systemic one, much like the data reported for the porcine models of VAP infected with *P*. *aeruginosa* [38, 40, 41]. Previous reports found that the inflammatory response was sustained for up to 96 h [38, 40], but we observed a very acute inflammatory response in the lungs that peaked 6 h after infection and decreased by 24 h. The pigs infected in our study were mechanically ventilated for only 20 min, unlike those used in the previous model of *P*. *aeruginosa* infection during VAP (tidal volume = 15 mL.kg-1, positive end-expiratory pressure = 0 cm H_2_O, respiratory rate = 15 breath.min-1, and inspiratory/expiratory ratio = 0.33, 21% oxygen in air. This procedure is associated with alterations in the immune response, cytokine release and infection [42].

We found that *P*. *aeruginosa* increased hyper-reactivity in the bronchi of pigs and the release of pro-inflammatory cytokines. These pro-inflammatory molecules play an important role in the response to a *P*. *aeruginosa* infection by regulating neutrophil trafficking from the blood to the inflamed tissues [43–45]. Interestingly, TNF-α level in BAL decreased significantly 24 p.i. despite the elevated number of inflammatory cells observed in the lung. This could be related to the induction of tolerance to the inflammatory stimuli as it has been previously observed in a mice model of repeated pulmonary LPS exposure [46]. The numbers of neutrophils in the differential WBC increased transiently (from 20% at 0 h to 60% at 6 h) and there was a massive influx of neutrophils into the lungs, despite the fact that the blood of pigs contains a smaller fraction of neutrophils (20–45%) than does human blood (40–80%). This event was associated with transient increases in all the neutrophil serine proteases and the secretion of NETs, even though they were barely detectable using BAL fluids, but quite readily detected in purified activated pig neutrophils [24], probably for mechanical reasons. Such an intense neutrophilic response is a hallmark of *Pseudomonas* lung infections in both CF and VAP [47, 48]. The efficacy of drugs targeting NSPs has presently been tested only in mice. Their results are difficult to interpret due to differences in the physicochemical properties and substrate specificities of mouse enzymes and those of human neutrophils [49, 50]. We have recently shown that human and porcine blood neutrophils and their proteases behave very similarly *in vitro* [24]. However, the neutrophil phenotype and responsiveness change once they are activated during migration from the peripheral blood to the airways [44, 51, 52] and lung secretions may alter the function and proteolytic potential of NSPs due to the presence of inhibitors. We have now demonstrated that the *in vivo* physicochemical properties and substrate specificities of pig lung NSPs are similar to those of humans. The experiments with the anti-peptide antibodies raised against human proteases confirmed the presence of all three NSPs at the surface of neutrophils and on the NETs of pigs. Immunoblotting analysis using anti-human protease antibodies indicated that the endogenous NSP inhibitors were overwhelmed: we detected both free proteases and complexed forms, as in human BAL fluids [53]. These data are especially important in animal models of lung diseases where neutrophils play a prominent role. For example, the pig model of CF develops lung abnormalities similar to those observed in humans, unlike the mouse model [54]. Hence, therapeutic inhibitors that target the active sites of human NSPs can be tested in the pig.

We conclude that inoculating pigs with *P*. *aeruginosa* produces a neutrophilic response similar to that seen in humans. The resemblance between the functions of pig and human neutrophils in their response to inhibitors, and the *in vivo* data presented here indicate that pigs are suitable candidates to model neutrophil-dependent lung inflammatory diseases such as CF.

## Supporting Information

S1 Fig Number of goblet cells in the tracheae of control and infected pigs. A. Samples collected 6 h p.i. B. Samples collected 24 h p.i. C. Mucous goblet cells in the trachea (x 400). Data are means ± S.E.M. ** indicates p<0.01.
(TIF)

S1 Table Health status of pigs. The pigs used for the study were obtained from a herd seronegative for the following common diseases.
(PDF)
