# Supplementary figures and images for: The Pig: A Relevant Model for Evaluating the Neutrophil Serine Protease Activities during Acute Pseudomonas aeruginosa Lung Infection
Source: PLoS One. 2016 Dec 16;11(12):e0168577. doi: 10.1371/journal.pone.0168577 (PMC5161375; doi:10.1371/journal.pone.0168577)

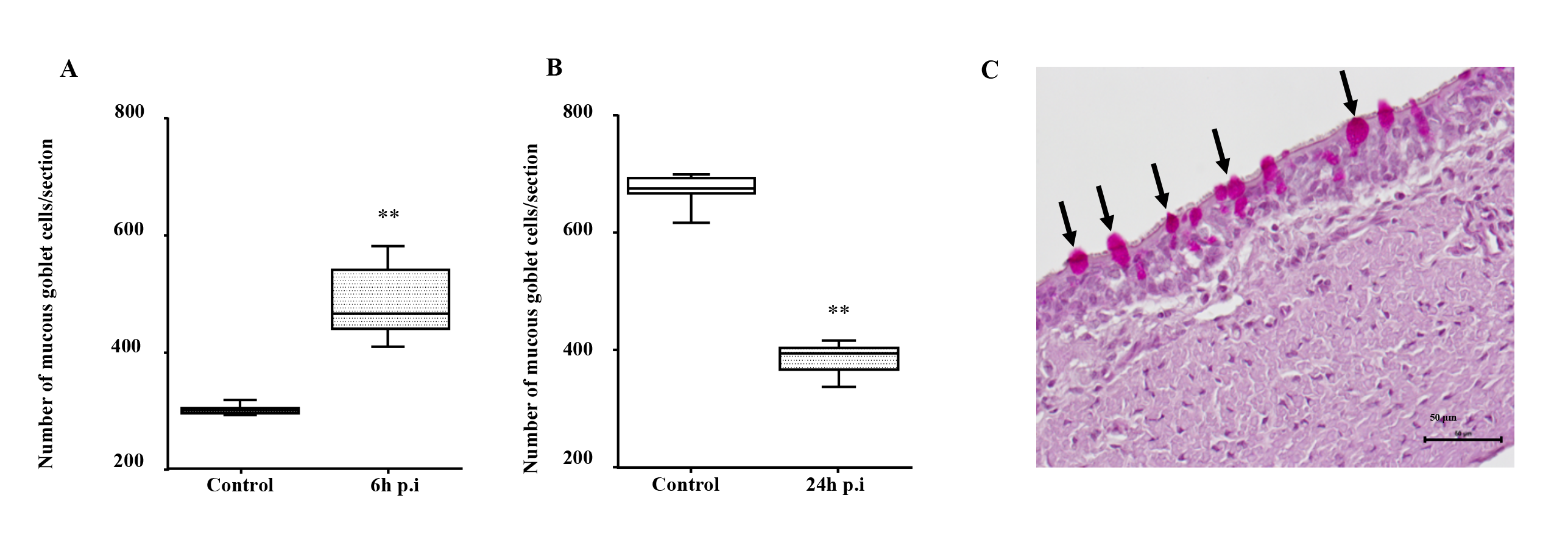

Supplement: S1 Fig — A. Samples collected 6 h p.i. B. Samples collected 24 h p.i. C. Mucous goblet cells in the trachea (x 400). Data are means ± S.E.M. ** indicates p<0.01. (TIF) [file pone.0168577.s001.tif]
