# Supplementary material for: The Pig: A Relevant Model for Evaluating the Neutrophil Serine Protease Activities during Acute Pseudomonas aeruginosa Lung Infection
Source: PLoS One. 2016 Dec 16;11(12):e0168577. doi: 10.1371/journal.pone.0168577 (PMC5161375; doi:10.1371/journal.pone.0168577)
Supplement: S1 Table — The pigs used for the study were obtained from a herd seronegative for the following common diseases. (PDF) [file pone.0168577.s002.pdf]

**S1 Table. Health status of pigs.** The pigs used for the study were obtained from a herd seronegative for the following common diseases.

| **Bacteria** | **Virus** |
|---|---|
| *Mycoplasma hyopneumoniae* | Hepatitis E virus |
| *Actinobacillus pleuropneumoniae* | Classical Swine Fever virus |
| *Bordetella bronchiseptica* | African Swine fever virus |
| *Pasteurella multocida* | Border Disease virus |
| *Haemophilus suis* | Aujeszky's disease virus |
| *Streptococcus suis de type 2* | Swine diarrhea virus |
| *Brachyspira hyodysentteriae* | Swine respiratory coronavirus |
| *Lawsonia intracellularis* | Porcine reproductive respiratory syndrome virus (PRRSV) |
| *Brucella suis* | Porcine Parvovirus (PPV) |
| *Listeria monocytogenes* | Influenza virus type A (H1N1, H1N2, H3N2 and pH1N1) |
| *Campylobacter spp.* | Porcine circovirus type 2 |
